# Low Adenosine Deaminase (ADA) Does Not Exclude Tuberculosis: A Case of Loculated Pleural Effusion Diagnosed by a Medical Thoracoscopic Pleural Biopsy

**DOI:** 10.7759/cureus.108617

**Published:** 2026-05-10

**Authors:** Somesh M Mashalkar, Anil Sontakke, Saood Ali, Yash Kumbhare, Prashant Ghule

**Affiliations:** 1 Respiratory Medicine, NKP Salve Institute of Medical Sciences &amp; Research Centre, Lata Mangeshkar Hospital, Nagpur, IND

**Keywords:** ada levels, anti-tuberculosis therapy, extrapulmonary tuberculosis (eptb), histopathologic diagnosis, medical thoracoscopy, pleural biopsy, pleural tuberculosis

## Abstract

Tubercular pleural effusion is a common cause of exudative pleural effusion, particularly in endemic regions, and adenosine deaminase (ADA) is widely used as a supportive diagnostic marker. However, lower-than-expected ADA levels can lead to diagnostic uncertainty. We report a 21-year-old male who presented with prolonged fever, weight loss, and dry cough. He was initially managed as a case of parapneumonic effusion due to lymphocytic exudative pleural fluid with low ADA levels. Persistent symptoms and imaging findings of multiple loculated pleural collections with associated lung collapse prompted further evaluation. Medical thoracoscopy revealed septations and nodular pleural lesions, and targeted biopsy demonstrated chronic granulomatous inflammation consistent with tuberculosis. This case underscores the limitations of interpreting ADA in isolation and highlights the importance of integrating clinical, radiological, and histopathological findings. Early use of a thoracoscopic pleural biopsy plays a key role in establishing diagnosis in complex or inconclusive pleural effusions.

## Introduction

Pleural effusion, defined as the accumulation of fluid within the pleural space, is a common clinical condition with a wide range of causes, including infections, malignancy, and systemic inflammatory diseases. Based on biochemical characteristics, pleural effusions are broadly classified as transudative or exudative, with exudative effusions reflecting increased capillary permeability due to inflammation or infection [1,2].

Tubercular pleural effusion is one of the most frequent causes of exudative effusion in regions such as India and remains an important global health problem [3,4]. It typically arises from a delayed hypersensitivity response to *Mycobacterium tuberculosis* antigens within the pleural space, resulting in lymphocyte-predominant inflammation and fluid accumulation.

Adenosine deaminase (ADA), an enzyme involved in purine metabolism, serves as a surrogate marker of T-cell activation and cell-mediated immunity. Pleural fluid ADA levels above 40 U/L are generally considered suggestive of tuberculosis [5,6]. However, ADA is not entirely reliable, and lower values may be observed in certain clinical scenarios, leading to diagnostic uncertainty if interpreted in isolation.

In such situations, further evaluation is warranted. Medical thoracoscopy, a minimally invasive procedure performed under local anesthesia, enables direct visualization of the pleural cavity and targeted biopsy of abnormal areas, thereby significantly improving diagnostic accuracy in undiagnosed exudative pleural effusions [7].

## Case presentation

A 21-year-old male presented with a one-month history of high-grade fever with chills, dry cough, loss of appetite, and significant weight loss. There was no prior history of tuberculosis, no known contact with a case of active tuberculosis, and no evidence of immunocompromised status. HIV testing was negative.

Fifteen days prior to presentation, he had been admitted to another center with similar complaints and underwent thoracentesis. Pleural fluid analysis at that time revealed a lymphocyte-predominant exudative effusion with low ADA levels, and he was managed as a case of parapneumonic effusion. However, his symptoms persisted, prompting further evaluation.

On examination, the patient was febrile but hemodynamically stable. Oxygen saturation was 96% on room air. Respiratory system examination revealed reduced chest expansion on the right side, stony dullness to percussion over the right hemithorax, and diminished breath sounds in the corresponding areas, consistent with pleural effusion.

Baseline laboratory investigations (Table 1) showed anemia and thrombocytosis. Repeat pleural fluid analysis demonstrated ADA of 25.62 U/L, lactate dehydrogenase (LDH) of 290 IU/L, and total protein of 5.1 g/dL, consistent with an exudative effusion based on Light's criteria [2]. The fluid was lymphocyte predominant, and the acid-fast bacilli (AFB) smear was negative (Table 1).

**Table 1:** Laboratory investigations at presentation

Parameter	Patient Value	Reference Range
Haemoglobin (Hb)	9.7 g/dL	13-17 g/dL
Total leukocyte count (TLC)	5000/mm³	4000-11,000 /mm^3^
Neutrophils	67%	40-70%
Lymphocytes	23%	20-40%
Platelet count	5.85 lakh/mm³	1.5-4.5 lakh/mm³
Pleural fluid ADA	25.62 U/L	<40 U/L (non-tubercular range)
Pleural fluid LDH	290 IU/L	Pleural fluid LDH >2/3 of the upper limit of normal (ULN) for serum LDH (exudative)
Pleural fluid total protein	5.1 g/dL	>3 g/dL (exudative)
Light’s criteria ratio	0.6	Pleural fluid protein/Serum protein > 0.5 (exudative)

**Figure 1:**
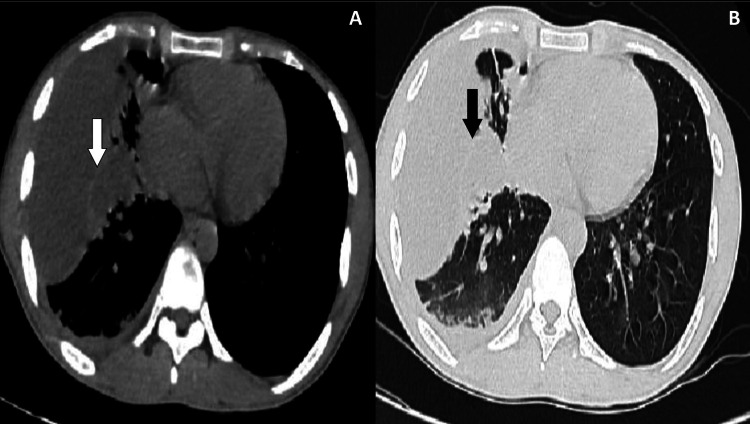
High-resolution computed tomography (HRCT) of the thorax showing loculated pleural effusion (A) Soft-tissue window: Axial HRCT image demonstrating a large right-sided pleural effusion with loculation. The white arrow indicates a loculated pleural fluid collection along the lateral aspect of the right hemithorax. (B) Lung window: Corresponding axial section showing loculated pleural effusion (black arrow) with underlying subsegmental collapse of the right lower lobe. The collapsed lung appears as increased parenchymal density adjacent to the effusion.

High-resolution computed tomography (HRCT) of the thorax revealed multiple loculated pleural fluid collections in the right hemithorax with associated subsegmental lung collapse (Figure 1), suggestive of a complex pleural effusion of infective etiology.

In view of persistent symptoms and inconclusive pleural fluid analysis, differential diagnoses considered included tubercular pleuritis, complicated parapneumonic effusion, and, less likely, malignancy.

**Figure 2:**
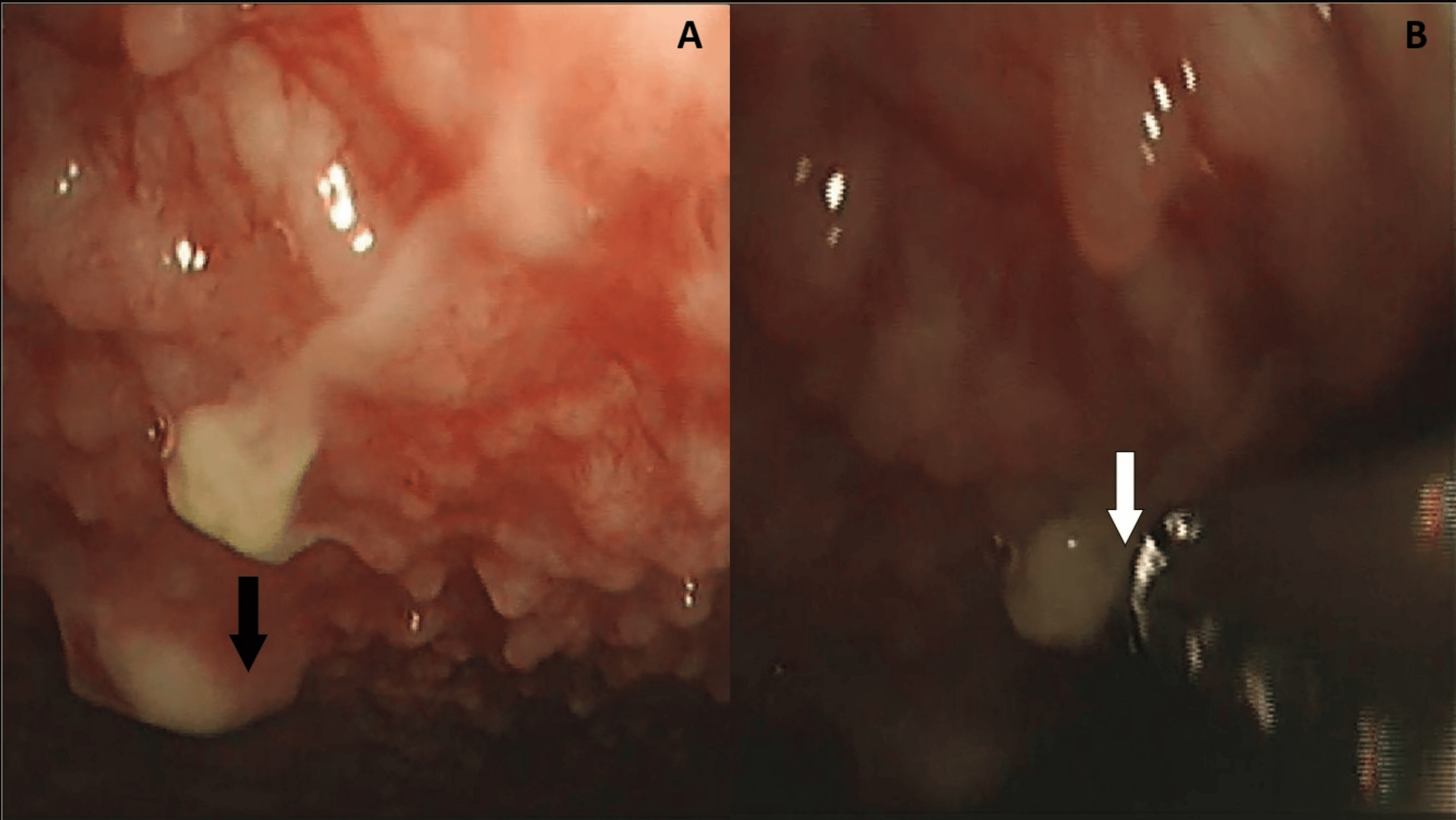
Thoracoscopic view of the pleural cavity showing nodular lesions (A) Thoracoscopic image of the parietal pleura obtained using a flexirigid pleuroscope. The surface appears irregular with multiple nodular projections and areas of hyperemia. The black arrow indicates a representative pleural nodule, which appears as a raised, pale-yellow lesion against the surrounding erythematous pleura. Adjacent areas show relatively smoother pleura for comparison, helping distinguish abnormal nodular regions from less involved surfaces. (B) Thoracoscopic pleural biopsy taken (white arrow) from similar area using biopsy forceps.

Medical thoracoscopy was subsequently performed under local anesthesia using a flexirigid pleuroscope (Figure 2). The procedure revealed multiple fibrous septations and nodular lesions over the parietal pleura. Multiple pleural biopsy samples were obtained from visually abnormal regions.

An intercostal drainage tube was placed with approximately 950 mL of total drainage. The drain was removed after output decreased to less than 10 mL over the three consecutive days.

**Figure 3:**
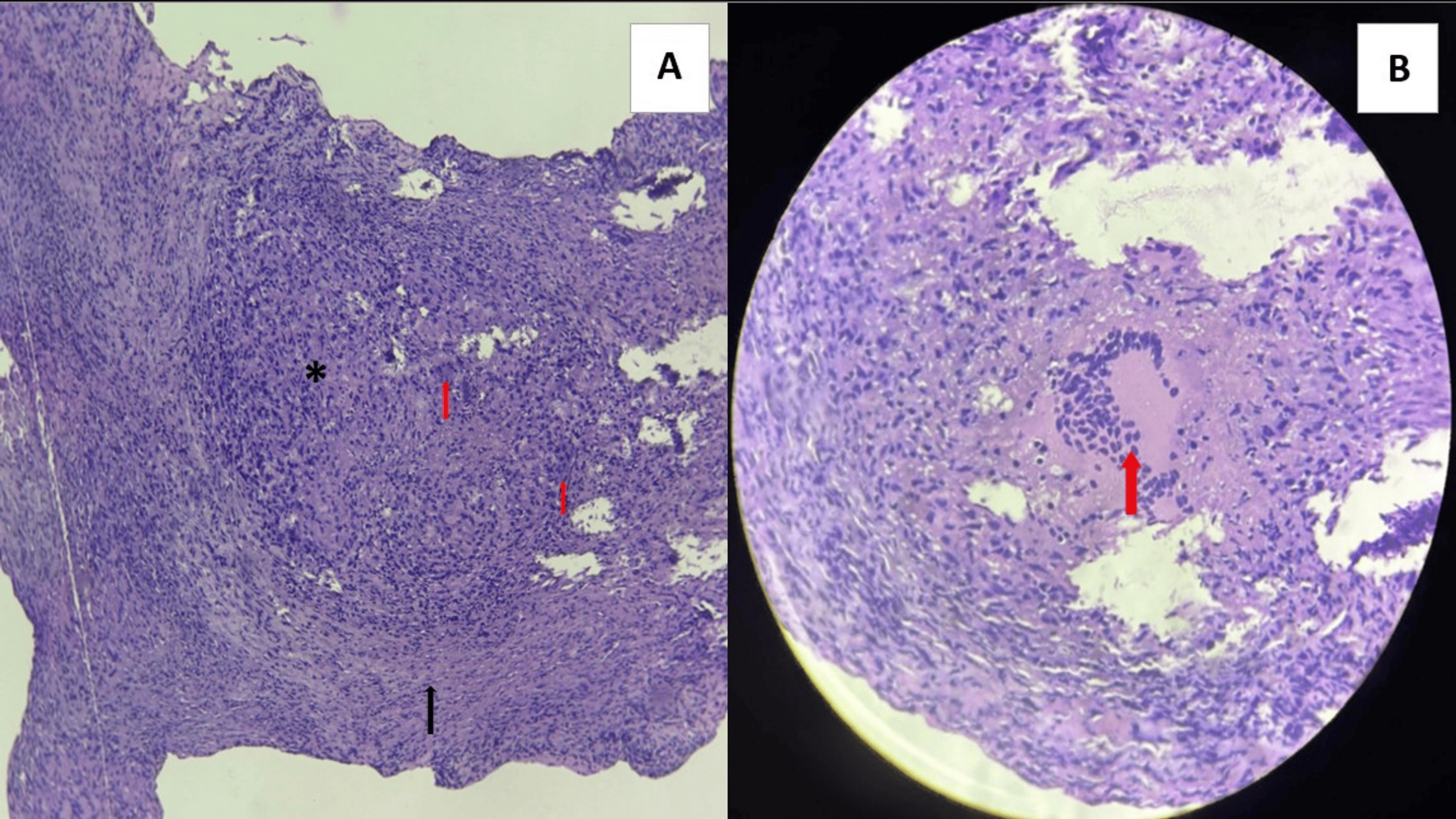
Histopathology of a pleural biopsy showing granulomatous inflammation (A) Thick fibrous tissue (black arrow), showing focal areas of granuloma composed of organized aggregates of activated macrophages called epithelioid cells (asterisk), lymphocytes, occasional giant cells (red arrows), and neutrophils, reflecting a chronic cell-mediated immune response (H&E, 40x). (B) Granuloma with epithelioid cells and multinucleated giant cells (red arrow). Giant cells are formed by fusion of macrophages and are commonly seen in granulomatous diseases such as tuberculosis (H&E, 400x).

Histopathological examination of the biopsy specimens demonstrated chronic granulomatous inflammation (Figure 3). In the appropriate clinical and radiological context, these findings were consistent with tubercular pleuritis.

A fixed-dose combination antitubercular therapy was started based on the above findings. Over the course of treatment, the patient showed improvement clinically and symptomatically.

## Discussion

ADA is widely used as a supportive diagnostic marker in tubercular pleural effusion, reflecting T-cell-mediated immune activation within the pleural space. However, its diagnostic reliability is not absolute and may be limited in certain clinical scenarios [5,6].

In the present case, the relatively low ADA level can be explained by several factors. The presence of a loculated pleural effusion may have resulted in sampling variability, as inflammatory activity can be unevenly distributed across different compartments. Additionally, early disease or variations in host immune response may lead to a less pronounced lymphocytic activation, thereby reducing ADA levels. Similar limitations of ADA interpretation have been highlighted in recent pleural disease reviews and tuberculosis literature [3,5].

A pleural fluid ADA threshold of approximately 40 U/L is commonly used to support a diagnosis of tuberculosis; however, false-negative results are well recognized [5,6]. The ADA value of 25.62 U/L in this patient, although below the conventional cut-off, falls within a range where tuberculosis cannot be reliably excluded, particularly when clinical and radiological findings are suggestive. Contemporary studies emphasize that ADA should be interpreted in conjunction with the clinical context rather than as a standalone diagnostic test [3,5].

The differential diagnosis of nodular pleural lesions includes tubercular pleuritis, malignant pleural disease, and complicated parapneumonic effusion. In this case, malignancy was considered less likely given the patient's young age, absence of a known primary tumor, and lack of imaging features suggestive of diffuse pleural malignancy. Similarly, the persistence of symptoms despite adequate antibiotic therapy made a purely parapneumonic process less probable. This structured clinical reasoning aligns with current pleural disease evaluation guidelines [7].

Microbiological confirmation of tubercular pleural effusion can be challenging. AFB smear has low sensitivity due to the paucibacillary nature of pleural fluid. Although nucleic acid amplification tests (such as GeneXpert) and mycobacterial cultures can improve diagnostic yield, their sensitivity remains variable and may be limited in pleural fluid samples [3,4]. Recent literature continues to emphasize the limited sensitivity of microbiological tests in pleural tuberculosis, reinforcing the importance of tissue diagnosis.

Radiological findings in this case, particularly the presence of multiple loculated pleural collections with associated lung collapse, raised suspicion for an organised infective process. These findings, combined with inconclusive pleural fluid analysis, warranted further evaluation with medical thoracoscopy. Thoracoscopy allows direct visualization of pleural surfaces and enables targeted biopsy, with a high diagnostic yield in undiagnosed exudative pleural effusions [7].

This case underscores the limitations of relying solely on biochemical markers such as ADA and highlights the importance of a comprehensive diagnostic approach. Integration of clinical presentation, imaging findings, and histopathological confirmation remains essential, with thoracoscopy serving as a key diagnostic modality in complex or inconclusive cases.

## Conclusions

This case highlights that low ADA levels do not exclude tubercular pleural effusion, particularly in the presence of compatible clinical and radiological findings. It underscores the importance of interpreting ADA within the broader clinical context rather than as a standalone diagnostic marker.

Medical thoracoscopy proved valuable in establishing the diagnosis by enabling direct visualization and targeted pleural biopsy in the setting of inconclusive pleural fluid analysis. While these observations reflect a single clinical experience, they support the role of thoracoscopic evaluation in complex pleural effusions where initial investigations are non-diagnostic.
